# Distribution and human‐caused mortality of Persian leopards *Panthera pardus saxicolor* in Iran, based on unpublished data and Farsi gray literature

**DOI:** 10.1002/ece3.5673

**Published:** 2019-09-27

**Authors:** Jamshid Parchizadeh, Mohammad Ali Adibi

**Affiliations:** ^1^ Tehran Iran; ^2^ Faculty of Environment and Energy Department of Habitats and Biodiversity Islamic Azad University Tehran Iran

**Keywords:** distribution, human‐caused mortality, Iran, *Panthera pardus saxicolor*, Persian leopard

## Abstract

Gray literature and data from unpublished sources can provide important scientific information that has not been published scientifically. The Persian leopard (hereafter leopard) *Panthera pardus saxicolor* is classed as endangered on the Red List of the International Union for Conservation of Nature and also is one of the least‐studied subspecies of leopard. It occurs in the Caucasus and Central and Southwest Asia. Iran contains more than 75% of the leopard's extant range, and the leopard population in this country serves as a source for neighboring countries. In this study, we determined the distribution and human‐caused mortality of leopards in Iran, by reviewing unpublished data and Farsi gray literature (which includes government reports) between 1 January 2010 and 30 December 2018. We created the most recent distribution map of the leopard in Iran. Our data display that human‐caused mortality of leopard in Iran mostly includes poaching and intentional poisoning, and roadkill.

## Graphical abstract

We created the most recent distribution map of the Persian leopards in Iran. We then identified poaching and intentional poisoning, and roadkill as the most important causes of leopard mortality in Iran.

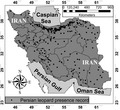

## 1 INTRODUCTION

The leopard *Panthera pardus* has the widest distribution of any large felid species and occurs across much of Africa (except the Sahara Desert) and Asia from the Middle East to the Pacific Ocean (Jacobson et al., 2016). The Persian leopard (hereafter leopard) *P. p. tulliana *(Valenciennes, 1856; 1,039), includes *ciscaucasica* and *saxicolor* (Kitchener et al., 2017), one of nine subspecies, is classed as endangered on the Red List of the International Union for Conservation of Nature with an estimated population size of 871 to 1,290 individuals (Stein et al., 2016). It occurs in some central and southwest Asian and Caucasian countries (Stein et al., 2016).

Iran contains more than 75% of leopard's extant range (Jacobson et al., 2016) and serves as a source for neighboring countries (Sanei et al., 2016). Iranian leopards are comprised of a monophyletic clade and are of the same maternal origin (Farhadinia et al., 2015). Their taxonomy as a distinct subspecies still remains obscure until further evaluations, but for the time being this subspecies is traditionally assigned to *P. p. saxicolor* (Yusefi, Faizolahi, Darvish, Safi, & Brito, 2019), as some studies investigating craniological patterns and molecular genetics showed that Iran is inhabited only by *P. p. saxicolor* (Farhadinia et al., 2015). According to unsubstantiated estimates, between 550 and 850 leopards are distributed within about 6,050 km^2^ in Iran, specifically in two great mountain ranges, the Zagros Mountains in the west and the Alborz Mountains in the north, which makes Iran the main stronghold of the leopard (Erfanian, Mirkarimi, Mahini, & Rezaei, 2013; Sharbafi, Farhadinia, Rezaie, & Braczkowski, 2016; Yusefi et al., 2019).

Gray literature is defined as material published and disseminated by governments, intergovernmental organizations, nongovernmental organizations, environmental consultancies, private companies, and freelance individuals that (a) is mostly made available on the Internet, (b) contains some useful data that is of lasting scientific value, and (c) is in addition to the scientific literature and not instead of it (Corlett, 2011). Similarly, unpublished data are original data from sources that have never been published. Here, we report leopard presence records across Iran obtained by reviewing unpublished data to create the most recent distribution map of the subspecies. Also, by obtaining mortality data from Farsi gray literature, we document human‐caused threats to the survival of leopards in Iran and propose some approaches to their conservation.

## 2 MATERIALS AND METHODS

We contacted 31 Provincial Offices of Iran's Department of the Environment (DoE; the governmental organization responsible for conserving Iran's fauna and flora) to obtain unpublished data on records of leopard presence between 1 January 2010 and 30 December 2018. According to the data sources, the presence points were recorded in the form of coordinates (i.e., longitudes and latitudes).

We used 1 January 2010 as our cutoff point, because we were searching for newer data compared to previous studies (Jacobson et al., 2016; Sanei et al., 2016).

We then used the Environmental Systems Research Institute Inc. (ESRI) ArcMap 10.3 (Release 10.3.1; ESRI) for Desktop Software to display leopard presence records in Iran.

We conducted a gray literature search on available Farsi Web sites and daily online newspapers using the equivalent search terms “leopard; equivalent Farsi word is 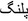 and “Persian leopard; equivalent Farsi word is 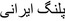 to access all relevant information about leopard mortality causes in Iran between 1 January 2010 and 30 December 2018. Then, we reviewed every source to avoid any data redundancy. We categorized mortality causes to unnatural, unknown, and natural, and then used unnatural causes for further analyses in our paper.

## 3 RESULTS

**Figure 1:**
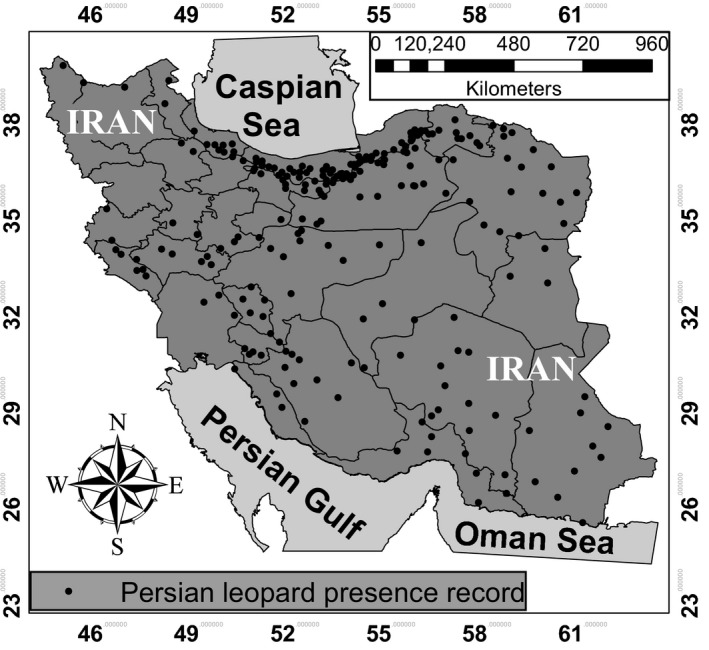
Map showing the records of Persian leopard presence across Iran between 1 January 2010 and 30 December 2018

**Figure 2:**
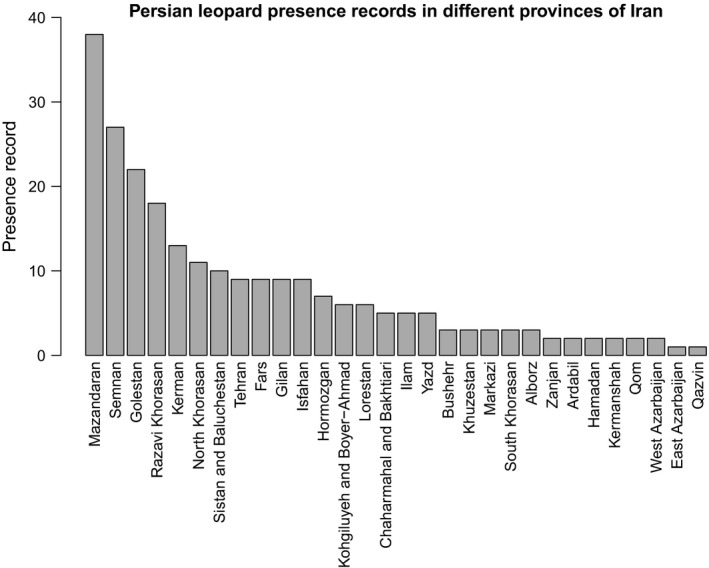
Number of Persian leopard presence records in 30 provinces of Iran between 1 January 2010 and 30 December 2018

By reviewing unpublished data between 1 January 2010 and 30 December 2018, we obtained 238 records of leopard presence (provided in Table S1). Leopards occurred in every province of Iran, except Kurdistan Province in western Iran (Figures 1 and 2). Mazandaran Province had the highest number of leopard presence records, followed by Semnan and Golestan Provinces (Figure 2, Table S1).

We reviewed 92 sources from Farsi gray literature and obtained the following results regarding the causes of mortality of leopards between 1 January 2010 and 30 December 2018.

**Figure 3:**
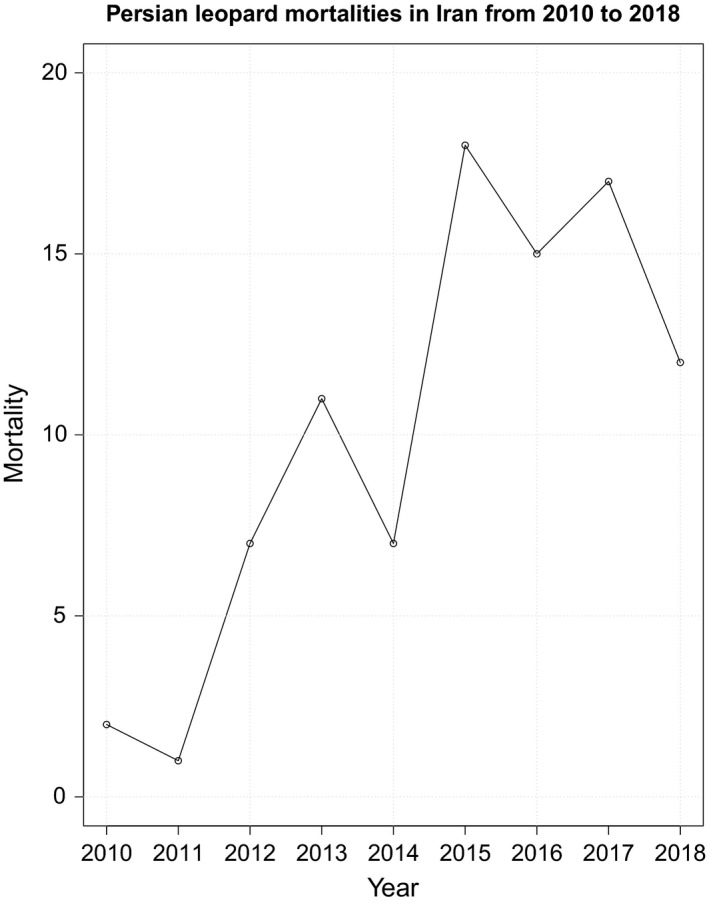
Number of Persian leopard mortalities recorded in Iran from 2010 to 2018

**Figure 4:**
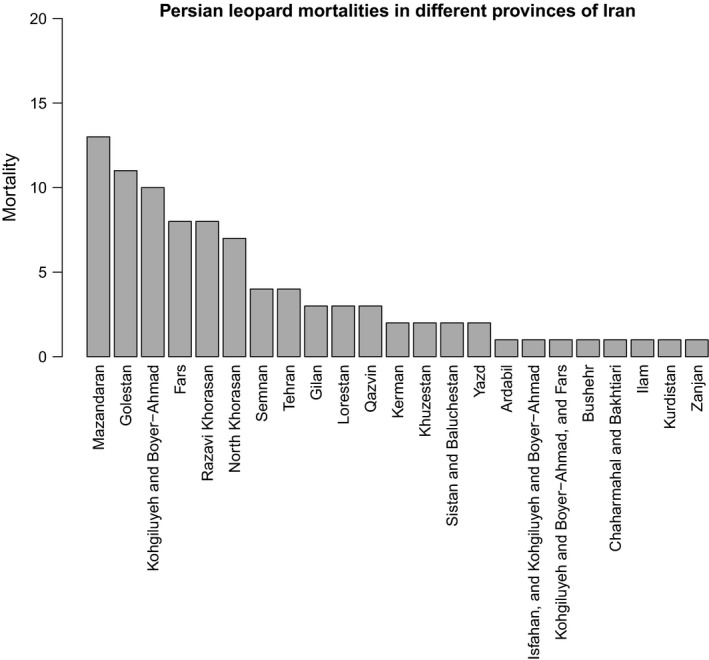
Number of mortality of Persian leopards recorded between 1 January 2010 and 30 December 2018 in 22 provinces of Iran

Of 90 leopard mortalities recorded in 22 provinces of Iran (provided in Table S2), unnatural mortality (i.e., shooting, stoning, trapping, intentional poisoning, hunting dogs, and roadkill) was 51%, unknown mortality was 33%, and natural mortality (i.e., old age, intraspecific or interspecific fights, drowning, snakebite, hunger, disease, and internal bleeding) was 16%. Our mortality data consisted of 40% unspecified gender, 31% females, and 29% males. The highest and the lowest number of leopard mortalities were recorded in 2015 and 2011, respectively (Figure 3). Mazandaran Province had the highest number of leopard mortalities, followed by Golestan, and Kohgiluyeh and Boyer‐Ahmad Provinces (Figure 4).

**Figure 5:**
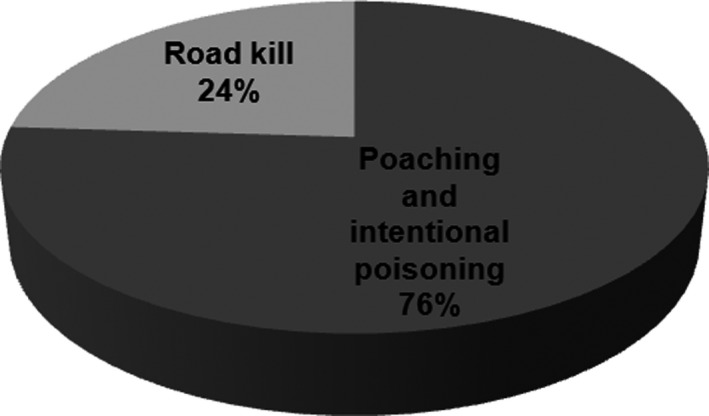
Human‐caused mortality of Persian leopard in Iran between 1 January 2010 and 30 December 2018

In unnatural mortality category, 76% were poached and intentionally poisoned, and the remaining 24% were killed in car accidents (Figure 5, Table S2). Mazandaran Province proved to have the highest rate of poaching with 26% (Table S2). Golestan Province showed the highest roadkill rate with 37% (Table S2).

## 4 DISCUSSION

According to our findings, Mazandaran Province had the highest number of both presence and human‐caused mortality records among other provinces during 2010–2018. This suggests that Mazandaran is a very important habitat to the leopard in Iran, and needs particular attention from both DoE in Tehran and Mazandaran Provincial Office of DoE. Although high number of presence records in Mazandaran province does not guarantee higher detection of leopards, yet they may be used as a baseline for planning practical conservation and management strategies in this province in the future. On the contrary, we did not document any leopard sign in Kurdistan Province between 2010 and 2018. In 2012, Kurdistan Provincial Office of DoE stated that three leopards were observed by local people in the Shahu‐Kusalan Protected Area (Iran's Department of the Environment, 2012), located between the towns of Sarvabad, Kamyaran, and Marivan (Hasti, Rouhi, Khodakarami, & Mahiny, 2016). However, camera traps failed to obtain any leopard photographs in this protected area (Iran's Department of the Environment, 2012). We received neither official confirmation nor data from Kurdistan Provincial Office of DoE, and as a result, we decided not to include these data to our database. Farhadinia, Johnson, Macdonald, and Hunter (2018) concluded that one of their collard leopards showed the largest range use in one year, expanding from Iran into Turkmenistan, resulting in an elongated range with 81.6 km. In addition, leopards have large home ranges with a mean home range of 103.4 ± *SE* 51.8 km^2^ (Farhadinia, Johnson, Macdonald, et al., 2018), and they occur in the mountainous areas of northeastern Iraq's Kurdistan region along the border with Iran and Turkey (Jacobson et al., 2016; Stein et al., 2016). Since the Shahu‐Kusalan Protected Area is located adjacent to Iraq's Kurdistan region, it is possible that the leopards have come from this area. Therefore, we recommend camera trap studies to determine whether this is the case. Sanei and Zakaria (2011) stated that leopard distribution in Iran's Kurdistan Province had declined severely. Yusefi et al. (2019) mentioned Kurdistan's name as one of the five Iranian provinces in which there were unconfirmed evidences of leopard presence. When the Iran–Iraq war broke out in the 1980's, military activities and the availability of weapons among local people increased (Sanei & Zakaria, 2011), which might be contributed to the reduction or extermination of the leopard population in this area of Iran.

We consider poaching and intentional poisoning, and roadkill, to be the main human‐caused threats to the leopard in Iran.

### 4.1 Poaching and intentional poisoning

Naderi, Farashi, and Erdi (2018) concluded that poaching and intentional poisoning of food eaten by leopards were the main causes of leopard fatalities in Iran. Sanei et al. (2016) concluded that almost 70% (*n* = 50) of the leopard mortalities during their study period resulted from poaching and poisoning. We found that 76% of the reported human‐caused leopard mortalities were due to poaching and intentional poisoning. One of the main reasons for human–leopard conflicts is linked to the extent to which leopards kill domestic stock and dogs (Farhadinia, Johnson, Hunter, & Macdonald, 2017). Livestock grazing is the main threat affecting large mammal distribution (Soofi et al., 2018), and diseases are major threats to livestock (Farhadinia et al., 2017) that make them vulnerable to leopard attacks, resulting in more human–leopard conflicts (Khorozyan, Soofi, Khaleghi Hamidi, Ghoddousi, & Waltert, 2015). The other reason for human–leopard conflicts, which has been overlooked in Iran for many years, is that leopards are killed for their skins. Huge profits can be made from trafficking luxury wildlife goods (Brashares et al., 2014), and due to a lack of official conservation measures in the forms of wildlife rangers and equipment (Ilam University, 2006; Sanei, 2007; Sanei et al., 2016; Sanei & Zakaria, 2011), the presence of poachers within most of Iran's protected areas remains as a threat to leopards (Sanei et al., 2016) and their main prey species (Ghoddousi et al., 2019). However, research is needed to determine the extent of this threat. An estimated minimum of 30–50 leopards are poached in Iran annually (Iranian Cheetah Society, 2011), some for their skins (Sanei, 2007). There have been several reports by the DoE indicating that the Environmental Protection Unit has discovered leopard skins in various places, including shops, houses, and markets (http://www.iew.ir). Leopard skins have been sold illegally for up to 270 million Rials (≈USD 2,077; USD 1 equals 130,000 Rials) in Iranian markets (Iran Environment & Wildlife Watch, 2015a) and USD 1,000 in markets in Arab countries (Khalaf‐von Jaffa, 2017). Therefore, the High Environment Council increased the fine from 50 million Rials (≈USD 385) to 800 million Rials (≈USD 6,154) and provided for a maximum of 3 years of incarceration for poachers who kill a leopard (Financial Tribune, 2015; Iran Environment & Wildlife Watch, 2015b). However, this strategy does not seem to be completely successful, because leopards are still poached. One reason may be the huge difference between the value of Rial and the value of the USD. Some poachers sell leopard skins to foreigners and receive USD. Therefore, it would still be profitable for them to sell in USD and pay the fine in Rials. Perhaps charging the fine in USD would help discourage poachers from killing leopards.

Poaching of leopard's main prey species has resulted in a lack of sufficient wild prey within some of Iran's protected areas (Iranian Cheetah Society, 2014; Sanei et al., 2016). The abundance of leopards is strongly dependent on the densities of their main prey species: wild goats *Capra aegagrus*, wild boar *Sus scrofa*, and urial sheep *Ovis vignei* (Ghoddousi et al., 2016; Reyahi Khoram, Rizvandy, & Reyahi Khoram, 2014). Leopards who suffer from a lack of sufficient wild prey, in addition to old and young dispersing leopards attack domestic dogs and livestock (Babrgir, Farhadinia, & Moqanaki, 2017; Farhadinia, Johnson, Hunter, & Macdonald, 2018). In response, rural residents and shepherds use both poisonous lures and guns to kill leopards to defend their livestock (Jacobson et al., 2016). The long‐term survival of the leopard is worrisome, given the ongoing escalated rate of human–leopard conflicts as an important threat (Iranian Cheetah Society, 2014), as the loss of every leopard is a major setback in efforts for safeguarding this subspecies (Maharramova et al., 2018). Therefore, the Ma Insurance Company (https://www.bimehma.com/), which is a for‐profit insurer, started contributing to leopard conservation free of charges by establishing a program to compensate for any losses of livestock and herder dogs inflicted by this predator in Iran (Sanei et al., 2016; The Tehran Times, 2017). In addition, the Ma Insurance Company contributed to the DoE up to 600 million Rials (≈USD 4,615) per leopard mortality caused by poaching, intentional poisoning, road collision, disease, drought, flood, fire, and herder dogs to reduce threats toward the leopard (Iran Environment & Wildlife Watch, 2016). However, conservation efforts were dealt a major blow in May 2019 when the Ma Insurance Company stopped insuring the leopard (The Islamic Republic News Agency, 2019). We propose that the DoE could reduce poaching of leopards and their prey by (a) increasing the number of wildlife rangers, as their current numbers are very low, (b) ensuring that wildlife rangers are provided with the necessary equipment, fuel, and training, and (c) developing educational programs, holding training workshops, and distributing literature highlighting the value of the leopard to raise awareness in local communities and general public, as low awareness is an important issue (Sanei et al., 2016).

### 4.2 Roadkill

Roadkill has been reported to be the second most important human‐caused threat to the leopard in Iran (Naderi et al., 2018; Sanei et al., 2016). Our results show that 24% of the leopards were killed in car accidents and Golestan Province showed the highest rate of road‐killed leopards (37%; 75% of them occurred in Golestan National Park [GNP]) among all provinces. A two‐lane highway named “Asiaei Highway” passes through GNP in Golestan Province, and numerous wildlife‐vehicle collisions, including the leopard road fatalities, occur on this highway annually (Dehghani Kazemi, Jafari, & Yavari, 2016; Safaei, Kahrom, & Mohammadi, 2012; Sanei et al., 2016). A new issue for leopards in Iran could be China's Belt and Road Initiative (BRI) and its deviating southern China–Pakistan Economic Corridor which cut through key landscapes of threatened large carnivores in west and central Asia, and largely overlaps with key habitats accommodating large populations of leopard in northern Iran (Farhadinia et al., 2019). Therefore, it is the sole responsibility of the Iran's Ministry of Roads and Urban Development (the governmental organization responsible for constructing roads) to save leopards’ lives by constructing wildlife crossing structures in the form of overpasses or underpasses associated with fencing in both GNP and BRI, before they are killed by vehicles.

## 5 CONCLUSIONS

By reviewing Farsi gray literature and unpublished data between 1 January 2010 and 30 December 2018, we obtained records that have allowed us to create the most recent distribution map of the leopard in Iran. From the documented human causes of mortality, we conclude that poaching and intentional poisoning, and roadkill are the main threats to leopards in Iran.

The Persian leopard is one of the least‐studied subspecies of leopard (Babrgir et al., 2017), and without taking active, targeted, and large‐scale conservation measures, it is in imminent danger of extinction (Khalaf‐von Jaffa, 2008; Khorozyan, Baryshnikov, & Abramov, 2006; Khorozyan, Malkhasyan, & Asmaryan, 2005). The Caucasian Leopard Working Group concluded that, without recovery of the Iranian populations (i.e., leopard and its prey), little hope is left for natural recolonization of the leopard across the Caucasus (Moqanaki, Breitenmoser, Kiabi, Masoud, & Bensch, 2013). In addition, Breitenmoser et al. (2014) concluded that not only does the high profile of leopard mean that it is a flagship for conservation in the region, but its ecological requirements, large areas of habitat and adequate prey, mean that efforts to restore the leopard will help conserve other threatened fauna and flora. Therefore, we suggest the DoE allocate necessary resources for conducting future surveys on the leopard populations in Iran and increase effective conservation measures, before it is too late.

## CONFLICT OF INTEREST

The authors declare no conflict of interest.

## AUTHOR CONTRIBUTIONS

Jamshid Parchizadeh designed the work, obtained the Persian leopard mortality data, analyzed and interpreted the data, prepared the figures, and wrote the draft and final versions of the manuscript. Mohammad Ali Adibi obtained the Persian leopard presence records and reviewed the manuscript.

## Data Availability

The authors agree to deposit their data in Dryad is https://doi.org/10.5061/dryad.pp281n6.
